# Acute Neurovascular Inflammatory Profile in Patients with Aneurysmal Subarachnoid Hemorrhage

**DOI:** 10.3390/biom15050613

**Published:** 2025-04-23

**Authors:** Ruby R. Taylor, Robert W. Keane, Begoña Guardiola, Raul Martí, Daniel Alegre, W. Dalton Dietrich, Jon Perez-Barcena, Juan Pablo de Rivero Vaccari

**Affiliations:** 1 Department of Neurological Surgery and The Miami Project to Cure Paralysis, University of Miami Miller School of Medicine, Miami, FL 33136, USAddietrich@med.miami.edu (W.D.D.); 2 Medical Scientist Training Program, University of Miami Miller School of Medicine, Miami, FL 33136, USA; 3 Department of Cellular Physiology and Molecular Biophysics, University of Miami Miller School of Medicine, Miami, FL 33136, USA; 4 Intensive Care Department, Son Espases University Hospital, 07120 Palma de Mallorca, Spain; 5 Neurosurgical Department, Son Espases University Hospital, 07120 Palma de Mallorca, Spain

**Keywords:** vascular inflammation, angiogenesis, blood–brain barrier, neurovascular unit, inflammation, aneurysm, biomarkers, subarachnoid hemorrhage

## Abstract

Aneurysmal subarachnoid hemorrhage (aSAH) is a life-threatening condition that results from intracranial aneurysm rupture, leading to the accumulation of blood between the arachnoid and pia mater. The blood breakdown products and damage-associated molecule patterns (DAMPs), which are released as a result of vascular and cellular compromise following aneurysm rupture, elicit local endothelial reactions leading to the narrowing of cerebral arteries and ischemia. In addition, vascular inflammation, characterized by activated endothelial cells, perpetuates disruption of the neurovascular unit and the blood–brain barrier. The uncertain prognosis of aSAH patients contributes to the necessity of a fluid biomarker that can serve as a valuable adjunct to radiological and clinical evaluation. Limited studies have investigated vascular inflammation and angiogenic protein expression following aSAH. Reliable markers of the vascular inflammatory and angiogenic response associated with aSAH may allow for the earlier detection of patients at risk for complications and aid in the identification of novel pharmacologic targets. We investigated whether vascular inflammatory and angiogenesis signaling proteins may serve as potential biomarkers of aSAH. Serum and cerebrospinal fluid (CSF) from fifteen aSAH subjects and healthy age-matched controls as well as hydrocephalus (CSF) no-aneurysm controls were evaluated for levels of vascular inflammatory and angiogenesis proteins. Protein measurement was carried out using electrochemiluminescence. The area under the curve (AUC) was calculated using receiver operating characteristics (ROC) to obtain information on biomarker reliability, specificity, sensitivity, cut-off points, and likelihood ratio. In addition, patients were grouped by Glasgow Outcome Score—Extended at 3 months post-injury to determine the correlation between vascular inflammatory protein levels and clinical outcome measures. aSAH subjects demonstrated elevated vascular inflammatory protein levels in serum and CSF when compared to controls. Certain vascular injury and angiogenic proteins were found to be promising biomarkers of inflammatory response in aSAH in the CSF and serum. In particular, elevated levels of serum amyloid-alpha (SAA) were found to be correlated with unfavorable outcomes following aSAH. Determination of these protein levels in CSF and serum in aSAH may be utilized as reliable biomarkers of inflammation in aSAH and used clinically to monitor patient outcomes.

## 1. Introduction

Aneurysmal subarachnoid hemorrhage (aSAH) is a life-threatening condition that results from intracranial aneurysm rupture, leading to the accumulation of blood between the arachnoid and pia mater [1,2,3]. Representing 5% of all strokes, aSAH occurs at an annual incidence in the USA of 6.9 to 9 in 100,000 cases [4]. Despite advances in neurocritical care and advanced imaging techniques, aSAH carries a 35% mortality rate, and more than 50% of survivors suffer from long-term disabilities [5,6,7]. Beyond primary brain injury, these patients also suffer from secondary complications, such as cerebral edema, hydrocephalus, increased intracranial pressure, and systemic cardiopulmonary complications [1,7,8,9]. Prognostication and realistic counseling on expected outcomes is of the utmost importance to patients and their relatives [10]. The grading systems proposed by Hunt and Hess as well as the World Federation of Neurological Societies have been widely used to aid in prognosis and guide treatment decisions in aSAH [11,12,13]. However, these scales have poor inter-rater variability and are limited in assessing secondary injury. Delayed cerebral ischemia (DCI) and cerebral infarction remain the top causes for morbidity and mortality following aSAH and have therefore been the focus of many studies up to this point [14,15,16,17]. Despite prevention methods and pharmacologic therapies targeting DCI, improvements in patient outcomes have not been observed, indicating that other pathophysiological processes must contribute to poor outcomes following aSAH [7,18,19]. In recent years, there has been an increase in interest in fluid biomarkers associated with brain damage in the course of neurological diseases [20,21,22,23,24], including traumatic brain injury [25,26,27,28], stroke [29,30,31], Alzheimer’s disease [32], and Parkinson’s disease. The uncertain prognosis of aSAH patients contributes to the necessity of fluid biomarkers that can serve as a valuable adjunct to radiological and clinical evaluation, providing increased insight into disease severity, progression, and resolution [33,34]. Furthermore, pathophysiological changes on the microscopic level induced by aSAH are often not visible on imaging but prove to be clinically significant in the long-term prognosis of patients. To date, no molecular biomarker has been validated in SAH using large prospective studies.

Current management of DCI is based on the restoration of cerebral perfusion by using induced hypertension or endovascular therapy with intra-arterial vasodilators and/or transluminal balloon angioplasty, but treatment efficacy is limited [35,36]. The only evidence-based strategy currently available for the prevention of DCI and the improvement in clinical outcomes remains the calcium channel blocker nimodipine, which has no significant effect on angiographic CVS [37,38].

The overall prognosis for patients with aSAH remains poor, with high rates of mortality and long-term disability. Rebleeding is prevented by securing the ruptured aneurysm with either surgical clipping or endovascular coiling, but the patient remains at risk for secondary neurological deterioration due to cerebral vasospasm (CVS) or other causes of DCI during the first 2 weeks following the acute bleeding event [39]. DCI is a clinical syndrome of neurological deterioration that is observed in approximately 30% of patients after aSAH. It can progress to cerebral infarction, which is a major predictor of cognitive impairment, quality of life deterioration, and poor long-term outcome [40].

Early brain injury (EBI) is a key determinant of poor prognosis following aSAH [41,42]. Blood–brain barrier (BBB) and microvascular architecture disruptions, as well as vascular inflammation, are hallmarks of EBI. The neurovascular unit (NVU), composed of vascular cells, glial cells, neurons, and extracellular matrix (ECM), contributes to maintaining BBB integrity [43,44]. The BBB is formed by endothelial cells, which are supported by astrocytes and pericytes [45,46]. Between endothelial cells, tight junction proteins and adherens junctions maintain the immune privilege status of the central nervous system (CNS) [45,47]. Cellular and molecular components of the NVU interact to ensure stable neuronal function, including reducing cell proliferation, protecting the CNS from exposure to toxins, and preventing inflammatory processes by regulating the passage of inflammatory cells [43]. Vascular inflammation, characterized by activated endothelial cells, perpetuates the disruption of the NVU in many CNS diseases [48,49]. BBB disruption is reported to occur within hours of aSAH and has been linked with downstream consequences [50]. Following aneurysm rupture, the accumulation of plasma proteins in the subarachnoid space leads to the degradation of tight junction proteins, transcellular channels, and endothelial retractions [43]. In addition, the accumulation of intravascular proteins in the brain interstitium results in increased intracranial pressure and brain volume [43]. In addition, blood breakdown products and damage-associated molecule patterns (DAMPs) released as a result of vascular and cellular compromise following aneurysm rupture elicit local endothelial reactions, leading to the narrowing of cerebral arteries and ischemia [41,51,52]. Injury also causes the upregulation of pro-inflammatory cell adhesion molecules, intercellular adhesion molecule 1 (ICAM-1), and vascular adhesion molecule 1 (VCAM-1), to induce leukocyte migration [48]. As a result of systemic inflammation, acute phase reactant proteins, C-reactive protein (CRP), and serum amyloid A (SAA) are also upregulated [48,53,54].

Following vascular injury and subsequent inflammation, angiogenic processes initiate the formation of new blood vessels [55]. Angiogenic growth factors, such as vascular endothelial growth factor (VEGF), induce the migration and proliferation of endothelial cells [55,56]. In the setting of aSAH, neurons, glial cells, and migrated macrophages release VEGF during cerebral ischemia. Platelet growth factor (PIGF) synergistically enhances VEGF activity [57]. Tyrosine kinase receptor, Tie-2, is responsible for maintaining vasculature in healthy and pathological states [55]. In addition, the resolution of brain edema occurs via lymphatic clearance of the brain [58]. VEGF-C is secreted by pro-inflammatory immune cells and binds to VEGFR-3 expressed on lymphatic vessels to stimulate lymphangiogenesis [58]. The growth factor basic fibroblast growth factor (bFGF) can increase tight junction protein expression to maintain vascular integrity, and it contributes to angiogenesis by enhancing the mitogenesis of endothelial cells [59,60].

Limited studies have investigated vascular inflammation and angiogenesis protein expression following aSAH. In comparison, aSAH is experienced by younger individuals when compared with Alzheimer’s disease, stroke, and dementia; therefore, the vascular injury that occurs in aSAH patients may have a unique molecular profile [50]. Reliable markers of the vascular inflammatory and angiogenic response associated with aSAH may allow for the earlier detection of patients at risk for complications and may aid in the identification of pharmacologic targets. In this study, we investigate the biomarker potential of vascular injury and angiogenesis proteins in the serum and cerebrospinal fluid (CSF) of aSAH patients compared to unaffected controls. The receiver operating characteristic (ROC) curve was determined for each potential biomarker. Furthermore, sensitivity and specificity were characterized and the area under the curve (AUC) was used to identify which inflammatory analytes have the greatest reliability and potential for clinical use as biomarkers. Furthermore, patients were grouped into favorable and unfavorable outcomes to analyze the contribution of vascular injury and angiogenesis protein expression to clinical outcomes in aSAH subjects. Finally, the proteins analyzed were utilized in a stepwise regression to predict clinical outcomes.

## 2. Materials and Methods

### 2.1. Patient Selection

This was a prospective and observational study in patients with aSAH who required an external ventricular drainage as part of their treatment. Patients with aSAH were recruited in Son Espases University Hospital (Palma de Mallorca, Spain). The Comité Ético de las Islas Baleares approved the study (IRB CEI IB 4914/22). Written informed consent was obtained from a family member or proxy according to the IRB. All subjects were admitted to the Neurological Intensive Care Unit at Hospital Universitario Son Espases, Palma de Mallorca, Spain. Patients included in the study met the following inclusion criteria: patients with aSAH, age 18 to 80 years, and ventriculostomy. Patients with past medical history relevant for CNS pathology, such as brain tumor, meningitis, cerebral vasculitis, or stroke, were excluded from this study.

Patients with aSAH received the following treatments (Table 1): GI prophylaxis with omeprazole (all the patients receive it); DVT prophylaxis with enoxaparin (all the patients receive it) after the aneurism was secured; vasospasm prophylaxis with nimodipine for 21 days (all the patients receive it); seizure prophylaxis with levetiracetam for 7 days (all the patients receive it). Other treatments included the following: analgesia: morphine, tramadol, acetaminophen, and dexketoprofen (NSAID) (not all patients received all treatments); sedation: propofol, midazolam (only for intubated patients); osmotherapy: 7.5% hypertonic saline (in case of intracranial hypertension); antibiotics (in case of infection); amoxicillin–clavulanate (at admission); vasoactive drugs: norepinephrine; insulin (for glycemic control).

### 2.2. Definitions

DCI was defined as a worsening of at least 2 points on the modified Glasgow Coma Scale (mGCS) or the abbreviated National Institutes of Health Stroke Scale (aNIHSS), lasting for at least 2 h, which cannot be entirely attributed to causes other than cerebral vasospasm. In all patients, neurological examinations were performed every 6 h per protocol. Cerebral vasospasm was defined as a narrowing of the cerebral arteries seen in the cerebral angiogram. Cerebral angiogram was performed for all patients on admission. The angiogram was also repeated if any patient presented a DCI that did not respond to hemodynamic treatment. New cerebral infarction was defined as the presence of new low-density areas on a computed tomography (CT) imaging scan of the brain. In this cohort of patients, a CT scan was performed for all patients on day 7 and on day 14 after the hospital admission.

### 2.3. Data Collection

Patients’ clinical data were recorded prospectively using the electronical medical records from the hospital (Power Chart; Millenium, 2011, Cerner Corporation, Kansas City, MS, USA). The 6-month outcome was assessed using the extended version of the Glasgow Outcome Scale (GOSE) [61]. The GOSE provides 8 categories of outcome: dead, vegetative state, lower severe disability, upper severe disability, lower moderate disability, upper moderate disability, lower good recovery, and upper good recovery. The 6-month GOSE was performed by a trained NICU attending (BG) by telephone consultation.

### 2.4. Sample Collection

Serum and cerebrospinal fluid (CSF) samples from patients with aSAH and samples from non-injured control donors were used in this study. Fifteen aSAH serum and CSF samples were obtained after informed consent. aSAH serum and CSF samples were obtained from 15 subjects (9 males (60%) and 6 females (40%)) with an age range of 36–77 years (median age of 64 years) (Table 2). Serum samples from healthy donors were purchased from BioIVT. The normal donor group consisted of samples obtained from 4 male and 4 female donors above age 55. Ten control CSF samples (Table S1) were obtained from patients with hydrocephalus admitted to Hospital Universitario Son Espases, Palma de Mallorca, Spain. Once the samples were obtained, they were immediately centrifuged at 2000× *g* for 10 min at 4 °C to pellet cellular bodies and debris. The supernatant was decanted and frozen at −80 °C until sample analysis. Samples analyzed were obtained at day 1 and day 5 post-aSAH. Outcome measurements were performed using the Glasgow Outcome Scale—Extended (GOS-E) at 3 and 6 months.

### 2.5. Electrochemiluminescence Immunoassay (ECLIA)

Vascular injury and angiogenesis biomarkers were measured using the MESO Quickplex SQ 120 instrument, Rockville, MD, USA. Serum and CSF levels were quantified using ECL detection in an array-based multiplex format. The vascular injury biomarkers CRP, ICAM-1, SAA, and VCAM-1 were measured using Meso Scale Discovery (MSD) V-PLEX Vascular Injury Panel 2 Human Kit (Cat. #K15198D, Meso Scale Diagnostics, Rockville, MD, USA). The angiogenesis biomarkers FGF, PIGF, Tie-2, VEGF, VEGF-C, VEGF-D, and Flt-1 were measured using Meso Scale Discovery (MSD) V-PLEX Angiogenesis Panel 1 Human Kit (Cat. # K15190G-1, Meso Scale Diagnostics, Rockville, MD, USA). Finally, the pro-inflammatory cytokines IFN-γ, IL-1β, IL-2, IL-4, IL-6, IL-10, IL-12p70, IL-17A, and TNF-α were measured using Meso Scale Discovery (MSD) S-Plex Proinflammatory Panel 1 Human Kit (Cat. # K15396S-1, Meso Scale Diagnostics, Rockville, MD, USA). Experiments were conducted according to the manufacturer’s instructions applying standard curves in duplicate on the 96-well MSD plate.

### 2.6. Statistical and Biomarker Analysis

Assay data were analyzed with PRISM 10 (GraphPad, Boston, MA, USA). The robust regression and outlier removal (ROUT) procedure (Q set to 1%) identified and removed outliers before further analysis was completed. Descriptive statistics were then obtained once normality was assured with the Shapiro–Wilk test or the D’Agostino and Pearson test. Multiple group comparisons were carried out using a Kruskal–Wallis test ANOVA or an ordinary one-way ANOVA to analyze the non-parametric and parametric data, respectively. The box plot shows the minimum and the maximum with all data points. A *p*-value of <0.05 indicated statistical significance. The AUC was calculated using the ROC to further characterize the biomarker potential and obtain information on specificity, sensitivity, cut-off points, and likelihood ratios. The cut-off point was defined as the highest likelihood of reliability vs. 1-specificity plot for each specific analyte. Higher sensitivity was prioritized over specificity to ensure a greater likelihood of reliability. The overall assay accuracy was measured, as well as the positive and negative predictive values and the Youden Index.

### 2.7. Regression Analyses

RStudio/RMarkdown (version 1.2.5033) was used for linear regression analysis to explain the contribution of vascular injury protein and pro-inflammatory cytokine levels as well as the inflammasome proteins analyzed in [62], to the Acute Physiology and Chronic Health Evaluation II (APACHE II), a predictor of ICU mortality. A stepwise regression procedure was completed using the vascular injury proteins and pro-inflammatory cytokines to predict APACHE II based on the lowest Akaike information criterion (AIC). Estimate, standard error, and *p*-values were then obtained for each of the predictors and intercepts of the linear regression. The Durbin–Watson (DW) statistic was used to evaluate the model for autocorrelation. Residuals, mean of residuals, root mean square error (RMSE), confidence intervals, and the Bayesian information criterion (BIC) for the best fit model were also calculated as described in [63].

## 3. Results

### 3.1. aSAH Patients Have Increased CSF Levels of Vascular Injury Proteins

**Figure 1:**
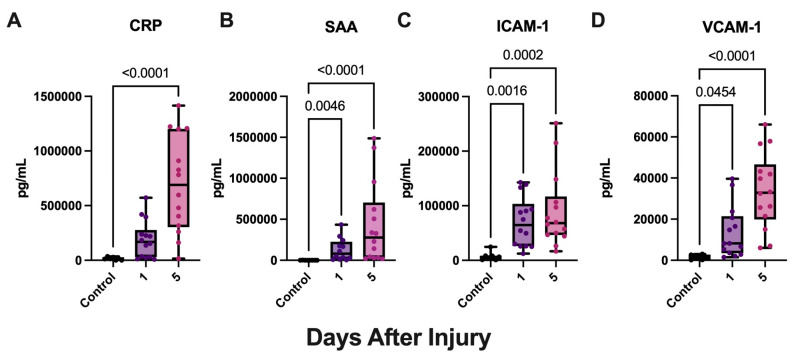
Vascular injury proteins are increased in the CSF of aSAH Subjects. ECLIA was used to quantify vascular injury proteins in the CSF of patients with aSAH and non-injured hydrocephalus controls. (**A**) CRP was significantly elevated at 5 days post-injury in patients with aSAH. Vascular injury proteins (**B**) SAA, (**C**) ICAM-1, and (**D**) VCAM-1 were significantly elevated at 1 and 5 days in patients with aSAH compared with hydrocephalus controls. (**A**) CRP: N: control: 7, aSAH: 14; (**B**) VCAM-1: N: control: 6, aSAH: 14; (**C**) ICAM-1: N: control: 7, aSAH: 14; (**D**) SAA: N: control: 8, aSAH: 12. Box plots and whiskers show the minimum, maximum, and all data points for each protein of interest with respective *p*-values listed above.

The acute phase protein CRP has been previously reported to be elevated in the venous blood and CSF of post-aSAH patients [64]. However, other vascular injury proteins as biomarkers of the inflammatory response following aSAH are yet to be elucidated. To determine the biomarker potential of vascular injury proteins in the tissue fluids of patients with aSAH, we evaluated the levels of vascular injury proteins, CRP, VCAM-1, ICAM-1, and SAA in the CSF of patients with aSAH and compared them to CSF samples from patients with hydrocephalus, which were used as non-aSAH controls. Using electrochemiluminescent immunoassays, we determined the levels of CRP (Figure 1A), SAA (Figure 1B), ICAM-1 (Figure 1C), and VCAM-1 (Figure 1D). aSAH patients demonstrated significantly higher levels of VCAM-1, ICAM-1, and SAA in the CSF for both timepoints when compared with hydrocephalus patient controls. In addition, aSAH patients demonstrated significantly higher levels of CRP on day 5 compared with controls. Together, this indicates that vascular injury protein levels are elevated in the CSF after aSAH.

### 3.2. aSAH Patients Have Increased Serum Levels of Vascular Injury Proteins

**Figure 2:**
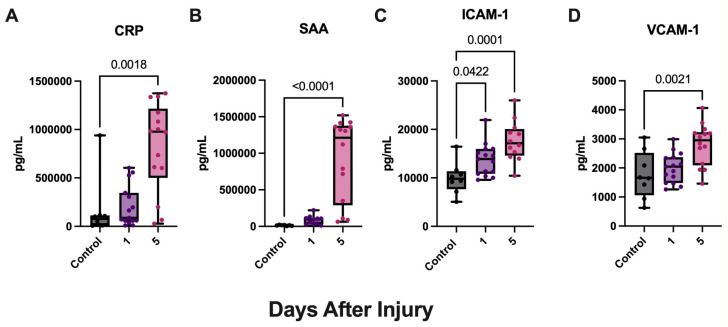
Vascular injury proteins are increased in the serum of aSAH subjects. ECLIA was used to quantify vascular injury proteins in the serum of patients with aSAH and healthy, age-matched controls. (**A**) CRP, (**B**) SAA, and (**D**) VCAM-1 were significantly elevated at 5 days post-injury whereas (**C**) ICAM-1 was significantly elevated at 1 and 5 days post-injury in patients with aSAH. (**A**) CRP: N: control: 8, aSAH: 15; (**B**) VCAM-1: N: control: 8, aSAH: 15; (**C**) ICAM-1: N: control: 8, aSAH: 15; (**D**) SAA: N: control: 7, aSAH: 11. Box plots and whiskers show the minimum, maximum, and all data points for each protein of interest with respective *p*-values listed above.

After determining vascular injury protein expression locally following aSAH, we aimed to investigate vascular injury protein expression systemically utilizing serum from the same patient cohort described above. aSAH patients demonstrated significantly higher levels of ICAM-1 (Figure 2C) for both timepoints when compared with age-matched controls. However, aSAH patients only demonstrated significantly higher levels of CRP (Figure 2A), SAAA (Figure 2B), and VCAM-1 (Figure 2D) on day 5 when compared with controls.

### 3.3. aSAH Patients Have Increased Levels of Pro-Inflammatory Cytokines in the Serum

**Figure 3:**
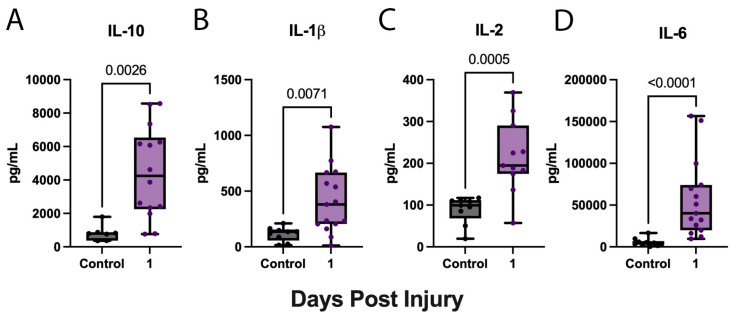
Pro-inflammatory cytokines are increased in the serum of aSAH subjects. ECLIA was used to quantify pro-inflammatory cytokines in the serum of patients with aSAH and healthy, age-matched controls. (**A**) IL-10, (**B**) IL-1β, (**C**) IL-2, and (**D**) IL-6 were significantly elevated 1 day post-injury in aSAH patients. (**A**) IL-10: N: control: 7, aSAH: 14; (**B**) IL-1β: N: control: 9, aSAH:15; (**C**) IL-2: N: control: 9, aSAH: 11; and (**D**) IL-6: N: control: 10, aSAH: 15. Box plots and whiskers show the minimum, maximum, and all data points for each cytokine of interest with respective *p*-values listed above.

We then used S-Plex MSD technology to determine the acute biomarkers of inflammation using a high-sensitivity assay. Accordingly, we determined the concentration of pro-inflammatory cytokines in the serum of aSAH patients at 1 day post-injury. aSAH patients presented significantly elevated levels of IL-10 (Figure 3A), IL-1β (Figure 3B), IL-2 (Figure 3C), and IL-6 (Figure 3D) for the first collection when compared to healthy, age-matched controls.

### 3.4. aSAH Patients Have Increased Levels of Angiogenesis Proteins in the CSF

**Figure 4:**
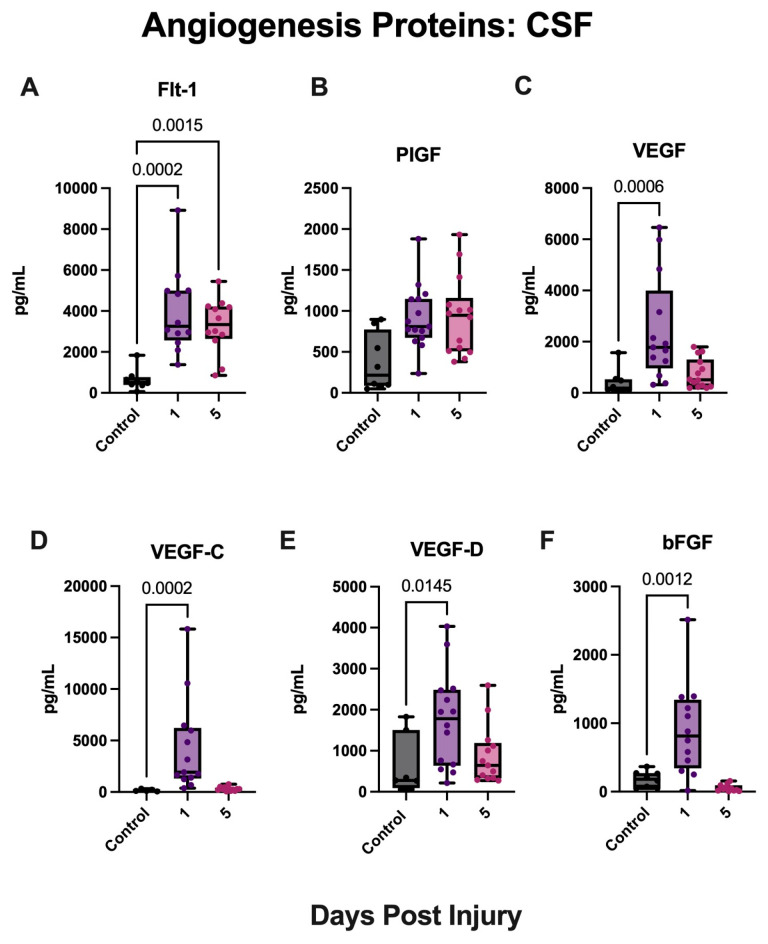
Angiogenesis proteins are increased in the CSF of aSAH subjects. ECLIA was used to quantify angiogenesis proteins in the CSF of patients with aSAH and non-injured hydrocephalus controls. Protein expression levels of (**A**) Flt-1, (**B**) PIGF, (**C**) VEGF, (**D**) VEGF-C, (**E**) VEGF-D, and (**F**) bFGF in aSAH pateints at 1 and 5 days post-injury. (**A**) Flt-1: N: control: 8, aSAH: 12; (**B**) PIGF: N: control: 8, aSAH:15; (**C**) VEGF: N: control: 8, aSAH: 13; (**D**) VEGF-C: N: control: 5 aSAH: 13; (**E**) VEGF-D: N: control:7 aSAH: 14; and (**F**) bFGF: N: control: 8 aSAH: 12. Box plots and whiskers show the minimum, maximum, and all data points for each protein of interest with respective *p*-values listed above.

Angiogenesis proteins have been previously utilized as blood-based biomarkers to monitor stroke recovery and evaluate therapeutic intervention [55]. To determine the biomarker potential of angiogenesis proteins in patients with aSAH, we evaluated the levels of angiogenesis proteins Flt-1, PIGF, Tie-2, VEGF, VEGF-C, VEGF-D, and bFGF in the CSF of the patient cohort and controls. aSAH patients presented significantly elevated levels of Flt-1 (Figure 4A) in the CSF at both timepoints when compared with hydrocephalus controls. Furthermore, aSAH patients demonstrated significantly elevated levels of VEGF (Figure 4C), VEGF-C (Figure 4D), VEGF-D (Figure 4E), and bFGF (Figure 4F) at day 5 when compared with controls. While aSAH patients demonstrated higher levels of PIGF for both timepoints, it was not a significant difference when compared to controls. Moreover, Tie-2 proteins were measured but were not sufficiently detected in the CSF.

### 3.5. aSAH Patients Have Increased Levels of Angiogenesis Proteins in the Serum

**Figure 5:**
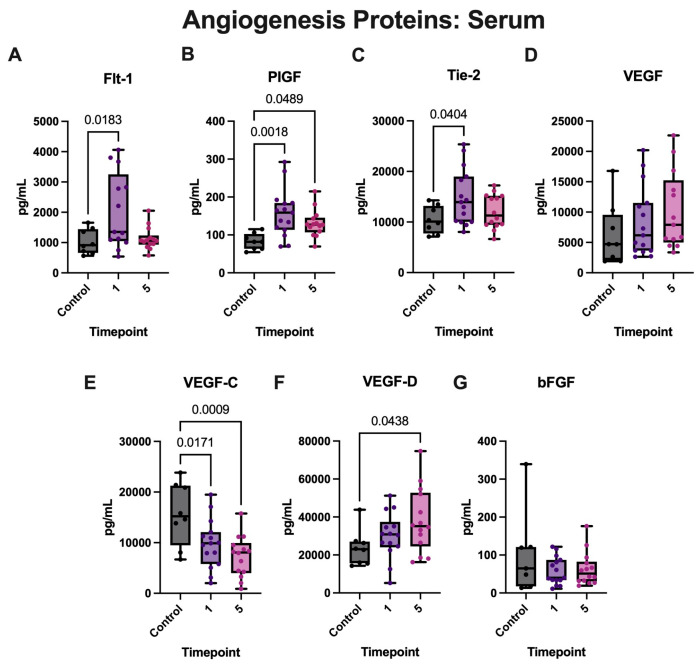
Angiogenesis proteins are increased in the serum of aSAH subjects. ECLIA was used to quantify angiogenesis proteins in the serum of patients with aSAH and healthy, age-matched controls. Protein expression levels of (**A**) Flt-1, (**B**) PIGF, (**C**) Tie-2, (**D**) VEGF, (**E**) VEGF-C, (**F**) VEGF-D, and (**G**) bFGF. Box plots and whiskers show the minimum, maximum, and all data points for each protein of interest with respective *p*-values listed above. (**A**) Flt-1: N: control: 8, aSAH: 13; (**B**) PIGF: N: control: 8, aSAH:15; (**C**) Tie-2: N: control: 8, aSAH: 15; (**D**) VEGF: N: control: 8, aSAH: 15; (**E**) VEGF-C: N: control: 8 aSAH: 15; (**F**) VEGF-D: N: control:8 aSAH: 14; and (**G**) bFGF: N: control: 7, aSAH: 15. Box plots and whiskers show the minimum, maximum, and all data points for each protein of interest with respective *p*-values listed above.

We then determined angiogenesis protein expression in the serum of aSAH patients. Accordingly, aSAH patients presented significantly elevated levels of PIGF (Figure 5B) at both timepoints when compared with healthy, age-matched controls. In addition, these patients demonstrated significantly elevated levels of Flt-1 (Figure 5A) and Tie-2 (Figure 5C) at day 1 and elevated levels of VEGF-D (Figure 5F) at day 5 when compared to controls. Patients did not demonstrate significantly elevated levels of VEGF (Figure 5D) when compared to controls. In addition, levels of VEGF-C (Figure 5E) and bFGF (Figure 5G) were higher in controls when compared to patients with aSAH.

### 3.6. Vascular Injury and Angiogenesis Proteins in CSF Are Reliable Biomarkers of aSAH

To determine whether vascular injury proteins are reliable biomarkers of aSAH in the CSF and serum, we plotted the receiver operating characteristics (ROC) curve for each protein and determined the area under the curve (AUC) for each timepoint (Table 3). While all proteins were plotted, we discuss proteins with an AUC > 0.9 as those are the most reliable biomarkers. Accordingly, in the CSF, SAA (Figure S1B) had the highest AUC for both timepoints at 1.000 (Table 3) with a specificity and sensitivity of 100% (Table 4). VCAM-1 (Figure S1A) also had high AUC values of 0.9286 and 1.000 (Table 3), at day 1 and day 5, respectively. However, the first timepoint of VCAM-1 demonstrated a lower specificity of 50% (Table 4). ICAM-1 (Figure S1C) demonstrated AUC values of 0.9898 for both timepoints, with a sensitivity of 100% and specificity of 85.71% (Table 4). Flt-1 (Figure S1D) had AUC values of 0.9896 and 0.9792 (Table 4) at day 1 and day 5, respectively, with a sensitivity of 100% and specificity of 85.71% (Table 4) for both timepoints. CRP (Figure S1E) presented a high AUC value of 0.9796 (Table 3) with a sensitivity of 100% and specificity of 71.43% for the 14th collection with a cut-off point of >14,909 pg/mL (Table 4). VEGF (Figure S1F) demonstrated an AUC value of 0.9135 (Table 4) with a sensitivity of 100% and specificity of 62.50% on day 1 with a cut-off point of >270.3 pg/mL (Table 4). VEGF-C (Figure S1G) had a high AUC value of 1.000 (Table 3) with a sensitivity and specificity of 100% on day 1 with a cut-off point of >351.2 pg/mL (Table 4). Overall, the results indicate that vascular injury proteins VCAM-1, SAA, ICAM-1, and CRP as well as the angiogenesis proteins Flt-1, VEGF, and VEGF-C are reliable CSF biomarkers of vascular inflammation following aSAH up to 5 days post-injury.

### 3.7. Vascular Injury and Angiogenesis Proteins in Serum Are Reliable Biomarkers of aSAH

SAA (Figure S2B) presented the highest AUC in the serum with a value of 1.000 (Table 5) with a sensitivity and specificity of 100% on day 5 and a cut-off point of >42,900 (Table 6). ICAM-1 had (Figure S2E) a high AUC value of 0.9271 (Table 5) with a sensitivity of 100% and specificity of 62.50% at a cut-off point of >10,331 pg/mL (Table 6). CRP (Figure S2C) demonstrated an AUC value of 0.9018 (Table 5) with a sensitivity of 85.71% and specificity of 80% on day 5 with a cut-off point of >172,486 pg/mL (Table 6). Further, PIGF demonstrated an AUC value of 0.9107 (Table 5) with a sensitivity of 92.86% and specificity of 75% on day 5 with a cut-off point of >94.53 pg/mL (Table 6). These results demonstrate that SAA, ICAM-1, and CRP are reliable biomarkers of vascular injury following aSAH in the serum up to 5 days post-injury. In addition, PIGF is a reliable biomarker of angiogenesis in aSAH patients 5 days post-injury.

### 3.8. SAA Is Elevated in the CSF of Patients with Unfavorable Outcomes After aSAH

**Figure 6:**
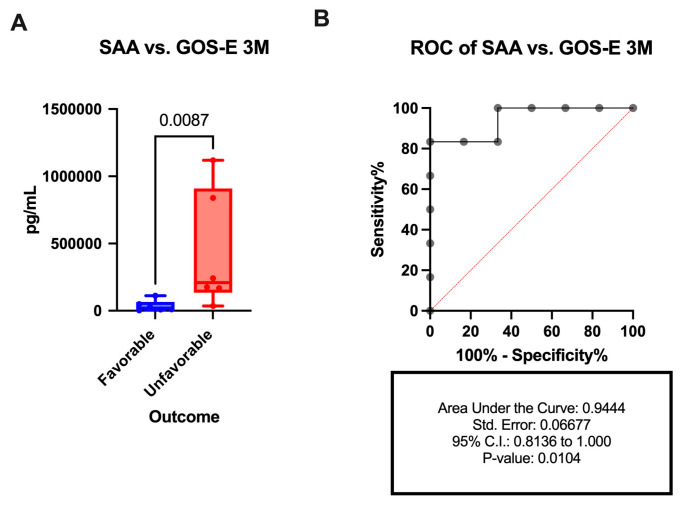
SAA as a prognostic biomarker of aSAH. Protein levels in pg/mL of (**A**) SAA and (**B**) associated ROC. Groups were divided into favorable and unfavorable outcomes based on the GOS-E. Box plots and whiskers show the minimum, maximum, and all data points for each protein of interest with respective *p*-values listed above. SAA: N = 6 favorable and 6 unfavorable.

We then separated aSAH patients according to their clinical outcomes. Patients were classified as favorable or unfavorable outcome based on their GOS-E at 3 months post-injury (GOS-E 3M) with patients with a score of 5 to 8 considered to have favorable outcomes, and those with a score of 1 to 4 considered to have unfavorable outcomes. Dichotomization into unfavorable and favorable outcomes was based on previous literature, and the well-established GOS-E scale, whereby patients with GOS-E < 5 are classified as dead or severely disabled and patients with GOS-E > 5 are classified as good recovery [26]. We found that SAA was significantly higher at 1 day post-injury in the CSF of patients with unfavorable outcomes (429,982 pg/mL +/−439,924 pg/mL) when compared to the CSF samples obtained from patients with favorable outcomes (35,375 pg/mL +/−41,055 pg/mL) (Figure 6A) with an AUC of 0.9444 (Figure 6B), suggesting that SAA is a reliable biomarker of outcomes after aSAH. The other vascular injury proteins VCAM-1, ICAM-1, and CRP were not statistically significant between outcome groups. 

### 3.9. Pro-Inflammatory Cytokines in Serum Are Reliable Biomarkers of aSAH

As previously described, we plotted the ROC curve to determine the AUC for the inflammatory cytokine panel analyzed (Table 7). IL-6 (Figure S3D) had the highest AUC for the first collection at 0.9733 (Table 7) with a sensitivity of 100% and specificity of 80% (Table 8). IL-2 (Figure S3C) presented a high AUC value of 0.9293 (Table 8) with a sensitivity of 90.91% and specificity of 100% (Table 8). Finally, IL-10 (Figure S3A) had an AUC value of 0.9082 (Table 7) with a sensitivity of 86.67% and specificity of 77.78% (Table 8). Taken together, these results indicate that the pro-inflammatory cytokines IL-10, IL-2, and IL-6 are reliable serum biomarkers of inflammation following aSAH at 1 day post-injury.

### 3.10. IL-6 and IL-2 Are Elevated in aSAH Patients with New Cerebral Infarcts

**Figure 7:**
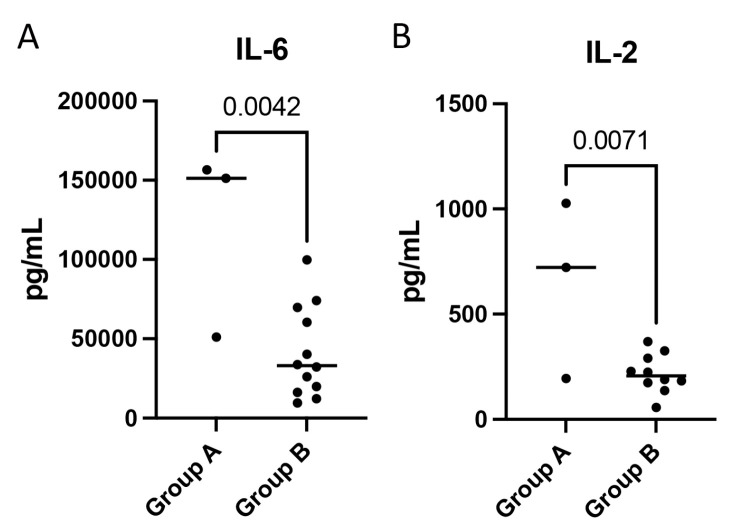
IL-6 and IL-2 are elevated in patients with new cerebral infarcts. Protein levels in pg/mL of (**A**) IL-6 and (**B**) IL-2. Group A comprised patients with new cerebral infarcts on day 7 or day 14 after admission as detected on CT. Group B patients did not demonstrate new cerebral infarcts. Box plots and whiskers show the minimum, maximum, and all data points for each protein of interest with respective *p*-values listed above. Group A: N = 3; Group B: N = 12.

aSAH patients were then dichotomized based on the presence of new cerebral infarcts on day 7 or day 14 after hospital admission. Patients in group A had new cerebral infarcts detected on CT imaging at day 7 or day 14 after hospital admission, while group B did not. We found that group A had significantly higher levels of IL-6 (Figure 7A) and IL-2 (Figure 7B) at 1 day post-injury in the serum when compared to group B. The other pro-inflammatory cytokines analyzed in this study were not statistically significant between groups. These data suggest that IL-6 and IL-2 may be good prognostic indicators in aSAH.

### 3.11. Linear Regression Analysis

To predict the contribution of previously described inflammasome proteins [62], vascular injury proteins, and pro-inflammatory cytokines to outcomes following aSAH, we conducted a multivariate linear regression that was fitted using a stepwise approach. The best model was determined by identifying the lowest AIC. Concentration of proteins/cytokines utilized in the model were measurements from one day post-aSAH. Following the stepwise method, the standard error, estimate (coefficients), and *p*-values for each of the inflammatory proteins and the intercept (slope) were calculated (Table 9). Additionally, based upon all identified inflammatory biomarkers, the BIC (74.0865), confidence intervals, DW autocorrelation, RMSE (2.230863), and mean of residuals (−8.723181 × 10^−17^) were determined for the best fit model:

Model: APACHE II ~ −1.39 × 10^1^ + 7.81 × 10^−5^ (SAA(CSF)) + 1.12 × 10^−1^ (IL-2 (Serum)) − 4.7 (Caspase-1(CSF)) − 6.15 × 10^−5^ (IL-6(Serum)) + 1.1 × 10^−3^ (IL- 10(Serum)) + 4.9 (IL-1β(CSF)) + 9.9 × 10^−2^ (IL-18(CSF))

## 4. Discussion

aSAH is a highly fatal and morbid disease with complex pathophysiology [4,65]. The surgical or endovascular repair of ruptured aneurysms in combination with pharmacologic management has improved outcomes in this patient population [66]. However, these interventions fail to reduce morbidity and mortality from secondary complications, such as delayed cerebral ischemia and non-neurologic medical complications [4,66]. Previously, attention was directed toward reducing cerebral vasospasm after aSAH. Clazosentan, an endothelin receptor antagonist, has been utilized to prevent cerebral vasospasm [18,45]. While it has been reported that clazosentan significantly reduces vasospasm in these patients, there were no significant improvements in functional outcomes or mortality rates [18]. Thus, there is a critical need for further investigation of the pathophysiological mechanisms contributing to poor prognosis in this patient population.

Following aneurysm rupture, inflammation is triggered by the release of blood into the subarachnoid space. Heme and other blood degradation products stimulate the inflammatory cascade and the upregulation of pro-inflammatory cytokines [67]. Beyond mechanical disruption of the BBB due to vessel rupture, inflammatory cytokines released during the acute response to hemorrhage cause the degradation of tight junction proteins (e.g., claudins and occludins) [68]. The combination of inflammation and BBB disruption is a central contributor to secondary brain injury in this patient population [45,69,70]. Thus, biomarkers of vascular injury have great potential to quantify the severity of vascular damage, inflammation, and BBB disruption, as well as contribute to the prediction of poor outcomes. Numerous studies have investigated the role of acute phase reactant proteins and adhesion proteins in the pathogenesis of cerebrovascular disorders [71,72]. Elevated CRP levels have been correlated with brain structural changes and the imaging markers of cerebral small vessel disease, as well as neurodegeneration [54]. CRP has also been previously investigated as a serum biomarker following aSAH. Patients with low GCS and high Hunt–Hess and Fisher grades have been shown to present with elevated serum CRP levels [73]. The cell adhesion molecules ICAM-1 and VCAM-1 have been shown to mediate leukocyte migration in aging and age-released degenerative disorders such as Alzheimer’s disease and vascular dementia [49]. Recent work has shown that SAA proteins can cross the intact BBB and impair BBB functions [74]. Angiogenic protein markers, such as VEGF and PIGF, have also been previously investigated. Accordingly, VEGF expression has been shown to remain elevated up to 90 days following stroke onset [55]. In a murine model of chronic hypoxia, PIGF-deficient mice exhibited a delayed angiogenic response, vascular dysfunction, and increased BBB permeability when compared to wild type mice [57]. While these studies have highlighted the importance of these proteins in the pathology of neurological diseases, the biomarker potential of these signaling proteins for prognostic purposes is yet to be elucidated, particularly in aSAH. Importantly, to perform a comprehensive biomarker study, it is necessary to determine the ROC curve as well as obtain the AUC value to determine biomarker reliability [75].

In this study, we used electrochemiluminescent immunoassays to measure vascular injury and angiogenesis proteins in the serum and CSF of 15 patients with aSAH. We identified significant increases in vascular injury proteins (CRP, SAA, ICAM-1, and VCAM-1) at 5 days post-injury in the serum and CSF when compared to healthy, age-matched and unaffected (hydrocephalus) controls, respectively. Elevated levels of these markers 5 days post-injury suggest an ongoing inflammatory response that may contribute to the pathophysiology of delayed neurological injury and systemic complications. Thus, monitoring the levels of these proteins may aid in assessing the severity of the inflammatory response and could be useful for prognostic purposes. Levels of the angiogenesis proteins VEGF, VEGF-C, VEGF-D, and bFGF were significantly elevated at 1 day post-injury in the CSF, while the angiogenesis protein Flt-1 was elevated up to 5 days post-injury in the CSF when compared to hydrocephalus controls. We also demonstrated that the angiogenesis proteins Flt-1 and Tie-2 were significantly elevated 1 day post-injury in the serum when compared to healthy, age-matched controls. In addition, PIGF was significantly elevated up to 5 days post-injury. Finally, VEGF-D significantly increased at 5 days post-injury. Previously, CSF was the primary source of novel biomarkers for CNS disorders due to its direct contact with the brain parenchyma and, therefore, its reflection of brain biochemical alterations following injury [76,77]. However, due to the invasive nature of CSF collection, there is high interest in less invasive fluid biomarker alternatives, such as peripheral blood.

To assess biomarker performance and reliability, we obtained ROC curves plotting biomarker sensitivity and specificity, as well as cut-off points that maximized sensitivity and specificity. Certain vascular injury and angiogenesis proteins demonstrated robust specificity and sensitivity and AUC values above 0.9, indicating they are reliable biomarkers of the vascular inflammatory response in aSAH. According to our analyses, CRP, SAA, ICAM-1, and VCAM-1 are reliable biomarkers of vascular inflammation in patients with aSAH when compared to controls. Both SAA and VCAM-1 presented an AUC > 0.9 for both timepoints measured. SAA demonstrated a high sensitivity and specificity of 100%. The angiogenesis proteins VEGF, VEGF-C, and Flt-1 also demonstrated an AUC > 0.9 with a robust sensitivity and specificity. Overall, the results indicated that these proteins are reliable CSF biomarkers of the vascular inflammatory and angiogenic response following aSAH. In the serum, SAA had the highest AUC value (1.000) on day 5 post-injury with a sensitivity and specificity of 100%. ICAM-1 and CRP also demonstrated high AUC values, 0.9271 and 0.9018, respectively, on day 5 post-injury. The angiogenic protein PIGF demonstrated an AUC value of 0.9107 with a high sensitivity and specificity on day 5. These results demonstrate that SAA, ICAM-1, CRP, and PIGF are reliable biomarkers of the vascular inflammatory and angiogenic response following aSAH, particularly at 5 days post-injury. By assessing serum as well as CSF biomarkers, we can analyze the vascular inflammatory and angiogenic response associated with aSAH on a local and systemic level. Moreover, the establishment of these proteins as serum biomarkers can have substantial clinical potential to provide prognostic information in a less invasive procedure.

We also investigated whether vascular inflammatory and angiogenic proteins are correlated with clinical outcomes in patients with aSAH. By dichotomizing patients into favorable and unfavorable outcomes using their GOS-E at 3 months post-injury, we found that patients with unfavorable outcomes (GOS-E < 5) exhibited significantly higher CSF levels of SAA when compared with patients with more favorable outcomes (GOS-E > 4). This finding suggests that high levels of SAA can serve as an indicator of poor outcome following aSAH. Moreover, we did not find significant levels of the other studied proteins between outcome groups.

Due to the low incidence of aSAH, the present proof-of-concept study consisted of a low sample size. Thus, future studies with increased patient numbers are necessary to validate the correlation between vascular inflammatory and angiogenic protein expression and clinical outcomes. Moreover, our results demonstrate the reliability of these proteins as biomarkers of vascular inflammation following aSAH. However, due to the small sample size, a limitation of the present study is that the cut-off points for each analyte/biomarker are not definite. Thus, further studies are needed with larger sample sizes in order to obtain a more definite AUC and the associated cut-off points with a more definite sensitivity and specificity for each potential biomarker. Ultimately, those cut-off points can be used for patient monitoring in the clinical setting. Moreover, future studies should also investigate the effects of therapeutics on these potential biomarkers in a larger population of affected individuals. Furthermore, a future study would be strengthened by using a cohort of individuals receiving the same therapeutic interventions after aSAH. In addition, patients with aSAH often experience chronic neurological dysfunction and cognitive decline in the months to years following injury. However, patients were lost to follow-up after 6 months, thus excluding the possibility of studying the relationship between chronic neurological deterioration and the vascular inflammatory biomarkers in the present study. Finally, future studies should carry out CSF and serum collection for longer than 5 days post-injury to examine whether these proteins correlate with secondary complications such as DCI and/or vasospasm.

Furthermore, we investigated the potential of inflammatory cytokines as biomarkers of aSAH. The serum levels of cytokines in patients with aSAH were assessed and compared with healthy, age-matched controls. This study revealed that the cytokines IL-10, IL-1β, IL-2, and IL-6 were significantly elevated following aSAH. Furthermore, IL-10, IL-2, and IL-6 demonstrated an AUC > 0.9 and were therefore determined to be reliable biomarkers of the inflammatory response following aSAH. Of note, previous studies have investigated the biomarker potential of similar inflammatory cytokines [69]. However, previous studies did not calculate the ROC and AUC, which are optimal for identifying cut-off points for biomarkers associated with the inflammatory response associated with aSAH.

DCI remains the leading cause of morbidity and mortality in this patient population, accounting for 23% of deaths and permanent neurological deficits in 37% of patients [78]. Previously, attention to mitigate DCI was directed toward reducing angiogenic vasospasm after aSAH. However, angiogenic therapies have not translated to improved outcomes. Interestingly, it was previously reported that the incidence of CVS was 67%, as demonstrated by angiography at 2 weeks post-injury in patients with aSAH; however, only 33% of these patients developed DCI [79]. Thus, there is a critical need for further investigation of the pathophysiological processes contributing to DCI and poor outcomes in this patient population [80]. Mechanisms of early brain injury that may contribute to DCI include inflammation, increased endothelin, decreased nitric oxide, oxidative stress, BBB breakdown, and cell death [81]. In this study, we found that IL-6 and IL-2 were elevated in aSAH patients who presented with new cerebral infarcts on CT imaging at 1 week or 2 weeks post-injury when compared to patients who did not demonstrate this finding. Thus, this study shines light onto IL-6 and IL-2 as potential targets contributing to the pathophysiology of aSAH complicated with cerebral infarct.

In this study, following vascular injury protein analyses and analyses of pro-inflammatory cytokines, a linear regression model was fitted to predict APACHE II, a classification score of disease severity in ICU patients, following aSAH. APACHE II has previously been described for use in predicting mortality in ICU patients; however, it is not routinely used in all hospitals [82]. Furthermore, the combination of APACHE II with inflammatory biomarkers has the potential to improve prognostication accuracy. Of the analytes utilized in the regression model, SAA in CSF presented the largest influence on increasing the APACHE II score, which is in alignment with our data that patients with higher SAA in CSF exhibit worse outcomes. Future studies with increased numbers of patients have the potential to increase the accuracy and significance of this model.

We also investigated the relationship between age and the expression of vascular injury and angiogenesis-related proteins contributing to aneurysm rupture. Age is a known risk factor for aneurysm formation and rupture, potentially influencing the biological response to vascular injury [83]. Furthermore, advanced age is a recognized prognostic indicator of poor outcome after aSAH [84]. To assess how vascular injury and angiogenic protein expression following aneurysm rupture is affected by age, we performed logistic regression analyses in which we accounted for age and biomarker expression for the presence or absence of aSAH. Accordingly, we identified significant associations for ICAM-1, FLT, and Tie-2 in the serum, whereas in CSF, ICAM-1, PIGF, and VEGF were significantly affected. Together, these findings indicate that the expression of vascular inflammatory biomarkers are also affected by age following aneurysm rupture. These findings underscore the importance of considering age-related biological variability when evaluating biomarkers and developing therapeutic strategies for aSAH.

Despite prevention methods and pharmacologic therapies targeting DCI, improvements in patient outcomes have not been observed, indicating that the pathophysiology of DCI remains poorly understood. Oral nimodipine remains the only pharmacological agent indicated for the prevention of DCI that has been shown to improve functional outcomes in aSAH [85]. A common theme in the research of preventative therapies is the use of promising drugs that have been shown to reduce the occurrence of CVS but ultimately did not improve functional outcomes in large, randomized studies. An example of this is the endothelin antagonist clazosentan, although this agent was recently approved in Japan [85]. For this reason, we must look for new therapeutic targets that prevent the occurrence of cerebral infarctions and improve the prognosis of aSAH patients, and we have conducted a search for potential new markers of the inflammatory response that may serve in the future as therapeutic targets to prevent the occurrence of DCI, vasospasm, or cerebral infarcts and thus improve the prognosis of aSAH patients.

## 5. Conclusions

Our study reveals that vascular inflammatory and angiogenic signaling proteins are reliable biomarkers of neuroinflammatory events associated with aSAH. The identification of signaling proteins after aSAH may help develop therapeutics to dampen the exacerbated inflammation after aSAH and to obtain prognostic information to monitor patient outcomes.

## Figures and Tables

**Table 1:** Treatments received by each patient.

Patient	Morphine	NSAID	Sedation	Vasoact.	Antibiot	Insulin
1	X	X				
2	X		X	X	X	X
3	X		X	X	X	
4	X	X		X		
5	X	X	X	X	X	X
6	X		X	X	X	
7	X		X	X	X	X
8	X		X	X	X	
9	X		X	X	X	
10	X	X			X	X
11	X	X	X		X	
12	X		X	X	X	
13	X	X	X		X	
14	X		X	X	X	X
15	X	X			X	

**Table 2:** Patient characteristics of subjects with aneurysm rupture.

Sex	Males	9 (60%)
	Females	6 (40%)
Age	Range	36–77
	Median	64
GCS	Average	9
	GCS 3	2 (13%)
	GCS 4	2 (13%)
	GCS 5	1 (6%)
	GCS 6	1 (6%)
	GCS 8	1 (6%)
	GCS 9	1 (6%)
	GCS 12	1 (6%)
	GCS 13	2 (13%)
	GCS 14	2 (13%)
	GCS 15	2 (13%)
WFNS scale	Average	3
	WFNS 1	2 (13%)
	WFNS 2	4 (27%)
	WFNS 4	4 (27%)
	WFNS 5	5 (33%)
Fisher scale	Average	4
	Fisher 3	1 (6%)
	Fisher 4	14 (94%)
ICU Days	Range	17–90
	Median	23
Hospital Days	Range	16–126
	Median	32
Patients with Hypertension		9 (60%)
Patients with Diabetes		2 (13%)
Patients with Delayed Cerebral Ischemia		2 (13%)

**Table 3:** ROC analysis results for CSF proteins.

CSF Protein/Collection	Area	STD Error	95% C.I.	*p*-Value
VCAM-1				
Day 1	0.9286	0.05650	0.8178 to 1.000	0.0030
Day 5	1.000	0.000	1.000 to 1.000	0.0005
SAA				
Day 1	1.000	0.000	1.000 to 1.000	0.0002
Day 5	1.000	0.000	1.000 to 1.000	0.0001
ICAM-1				
Day 1	0.9898	0.01640	0.9577 to 1.000	0.0003
Day 5	0.9898	0.01640	0.9577 to 1.000	0.0003
CRP				
Day 1	0.8673	0.07820	0.7141 to 1.000	0.0072
Day 5	0.9796	0.02595	0.9287 to 1.000	0.0005
Flt-1				
Day 1	0.9896	0.01678	0.9567 to 1.000	0.0003
Day 5	0.9792	0.02681	0.9266 to 1.000	0.0004
PIGF				
Day 1	0.8417	0.09479	0.6559 to 1.000	0.0081
Day 5	0.8571	0.08386	0.6928 to 1.000	0.0063
VEGF				
Day 1	0.9135	0.06251	0.7909 to 1.000	0.0018
Day 5	0.7589	0.1159	0.5319 to 0.9860	0.0478
VEGF-C				
Day 1	1.000	0.000	1.000 to 1.000	0.0014
Day 5	0.6833	0.1349	0.4190 to 0.9477	0.2463
VEGF-D				
Day 1	0.8367	0.09402	0.6525 to 1.000	0.0138
Day 5	0.7033	0.1453	0.4186 to 0.9880	0.1427
bFGF				
Day 1	0.8750	0.08616	0.7061 to 1.000	0.0055
Day 5	0.8523	0.08998	0.6759 to 1.000	0.0105

**Table 4:** Cut-off points for CSF proteins.

CSF Protein/Collection	Cut-Off Point (pg/mL)	Sensitivity (%)	Specificity (%)	Youden Index	LR	PPV	NPV	Accuracy (%)
VCAM-1								
Day 1	>1299	100	50	0.50	2.000	82	100	85
Day 5	>4524	100	100	1		100	100	100
SAA								
Day 1	>3055	100	100	1		100	100	100
Day 5	>10,091	100	100	1		100	100	100
ICAM-1								
Day 1	>10,360	100	85.71	0.86	7.000	93	100	95
Day 5	>12,498	100	85.71	0.86	7.000	93	100	95
CRP								
Day 1	>18,894	85.71	71.43	0.57	3.000	86	71	81
Day 5	>14,909	100	71.43	0.71	3.500	88	100	90
Flt-1								
Day 1	>1097	100	87.50	0.87	8.000	92	100	95
Day 5	>835	100	87.50	0.87	8.000	92	100	95
PIGF								
Day 1	>565	93.33	75	0.68	3.733	87	86	87
Day 5	>349	100	62.50	0.63	2.667	82	100	86
VEGF								
Day 1	>270.3	100	62.50	0.63	2.667	81	100	86
Day 5	>159.3	100	50	0.50	2.000	78	100	82
VEGF-C								
Day 1	>351.2	100	100	1		100	100	100
Day 5	>155.3	75	60	0.35	1.875	82	50	71
VEGF-D								
Day 1	>407.2	92.86	71.43	0.64	3.250	87	83	86
Day 5	>270.2	100	42.86	0.43	1.750	76	100	80
bFGF								
Day 1	>226.5	91.67	62.50	0.54	2.444	79	83	80
Day 5	<158.6	100	62.50	0.63	2.667	79	100	84

**Table 5:** ROC analysis results for serum proteins.

Serum Protein/Collection	Area	STD Error	95% C.I.	*p*-Value
VCAM-1				
Day 1	0.6000	0.1425	0.3208 to 0.8792	0.4386
Day 5	0.8571	0.0063	0.6984 to 1.000	0.0063
SAA				
Day 1	0.9481	0.05482	0.8406 to 1.000	0.0018
Day 5	1.000	0.000	1.000 to 1.000	0.0003
ICAM-1				
Day 1	0.8000	0.1100	0.5843 to 1.000	0.0201
Day 5	0.9271	0.06023	0.8090 to 1.000	0.0016
CRP				
Day 1	0.6667	0.1203	0.4309 to 0.9025	0.1967
Day 5	0.9018	0.06684	0.7708 to 1.000	0.0021
Flt-1				
Day 1	0.7308	0.1139	0.5075 to 0.9540	0.0822
Day 5	0.6161	0.1406	0.3405 to 0.8916	0.3749
PIGF				
Day 1	0.8917	0.06702	0.7603 to 1.000	0.0024
Day 5	0.9107	0.06121	0.7907 to 1.000	0.0017
TIE-2				
Day 1	0.7583	0.1019	0.5586 to 0.9580	0.0454
Day 5	0.6518	0.1188	0.4188 to 0.8847	0.2459
VEGF				
Day 1	0.6333	0.1302	0.3782 to 0.8864	0.3017
Day 5	0.7115	0.1202	0.4760 to 0.9470	0.1111
VEGF-D				
Day 1	0.6964	0.1170	0.4672 to 0.9257	0.1332
Day 5	0.7946	0.1022	0.5944 to 0.9949	0.0243

**Table 6:** Cut-off points for serum proteins.

Serum Protein/Collection	Cut-Off Point (pg/mL)	Sensitivity (%)	Specificity (%)	Youden Index	LR	PPV	NPV	Accuracy (%)
VCAM-1								
Day 1	>1448	86.67	37.50	0.24	1.387	72	60	70
Day 5	>1803	92.86	62.50	0.55	2.476	81	83	82
SAA								
Day 1	>15,788	90.91	57.14	0.48	2.121	77	80	78
Day 5	>42,900	100	100	1		100	100	100
ICAM-1								
Day 1	>10,288	86.67	62.50	0.49	2.311	81	71	78
Day 5	>10,331	100	62.50	0.63	2.667	80	100	85
CRP								
Day 1	>55,800	80	50	0.3	1.600	75	57	70
Day 5	>172,486	85.71	87.50	0.73	6.857	92	78	86
Flt-1								
Day 1	>973.2	84.62	62.50	0.47	2.256	79	71	76
Day 5	>950.2	78.57	62.50	0.41	2.095	79	62	63
PIGF								
Day 1	>94.88	86.67	75	0.62	3.467	87	75	83
Day 5	>94.53	92.86	75	0.68	3.714	87	86	86
TIE-2								
Day 1	>9874	86.67	50	0.37	1.733	76	67	74
Day 5	>9906	64.29	50	0.14	1.286	69	44	59
VEGF								
Day 1	>2624	100	37.50	0.38	1.600	75	100	78
Day 5	>5133	76.92	62.50	0.39	2.051	77	62	71
VEGF-D								
Day 1	>23,297	78.57	62.50	0.41	2.095	79	62	73
Day 5	>26,389	78.57	75	0.54	3.143	85	67	77

**Table 7:** ROC analysis results for serum pro-inflammatory cytokines at 1 day post-injury.

Serum Protein/Collection	Area	STD Error	95% C.I.	*p*-Value
IL-10	0.9082	0.06722	0.7764 to 1.000	0.0028
IL-1β	0.8519	0.08282	0.6895 to 1.000	0.0046
IL-2	0.9293	0.06963	0.7928 to 1.000	0.0012
IL-6	0.9733	0.02681	0.9208 to 1.000	<0.0001

**Table 8:** ROC analysis results for serum pro-inflammatory cytokines at day 1.

CSF Protein/Collection	Cut-Off Point (pg/mL)	Sensitivity (%)	Specificity (%)	Youden Index	LR	PPV	NPV	Accuracy (%)
IL-10	>1891	85.71	100	0.86		100	78	90
IL-1β	>154.5	86.67	77.78	0.64	3.900	87	78	84
IL-2	>126.9	90.91	100	0.91		100	90	95
IL-6	>7803	100	80	0.80	5.000	88	100	92

**Table 9:** Multivariate analysis for linear regression.

Variable	Coefficient (β_1_)	Standard Error	t-Statistic	*p*-Value
Intercept (β_o_)	−1.389 × 10^1^	3.607	−3.851	0.008451
SAA (CSF)	7.807 × 10^−5^	8.056 × 10^−6^	9.691	6.93 × 10^−5^
IL-2 (Serum)	1.121 × 10^−1^	1.086 × 10^−2^	10.323	4.83 × 10^−5^
Caspase-1 (CSF)	−4.609	7.375 × 10^−1^	−6.250	0.000778
IL-6 (Serum)	−6.148 × 10^−5^	2.064 × 10^−5^	−2.979	0.024672
IL-10 (Serum)	1.052 × 10^−3^	3.896 × 10^−4^	2.702	0.035504
IL-1β (CSF)	4.887	1.051	4.649	0.003058
IL-18 (CSF)	9.914 × 10^−2^	4.336 × 10^−2^	2.287	0.062237
R-squared	0.9611			
Adjusted R-Squared	0.9157			
BIC	74.0865			
Durbin–Watson	0.1489239			

## Data Availability

The original contributions presented in this study are included in the article/supplementary material.

## Supplementary Materials

The following supporting information can be downloaded at: https://www.mdpi.com/article/10.3390/biom15050613/s1, Figure S1: ROC for Vascular Injury and Angiogenesis Biomarkers of aSAH in CSF; Figure S2: ROC for Vascular Injury and Angiogenesis Biomarkers of aSAH in Serum. Figure S3: ROC for Pro-Inflammatory Cytokines of aSAH in Serum.
