# Supplementary material for: Acute Neurovascular Inflammatory Profile in Patients with Aneurysmal Subarachnoid Hemorrhage
Source: Biomolecules. 2025 Apr 23;15(5):613. doi: 10.3390/biom15050613 (PMC12108773; doi:10.3390/biom15050613)
Supplement: Supplementary file 1 [file biomolecules-15-00613-s001.zip › biomolecules-3560281-supplementary.pdf]

ROC CURVES: CSF

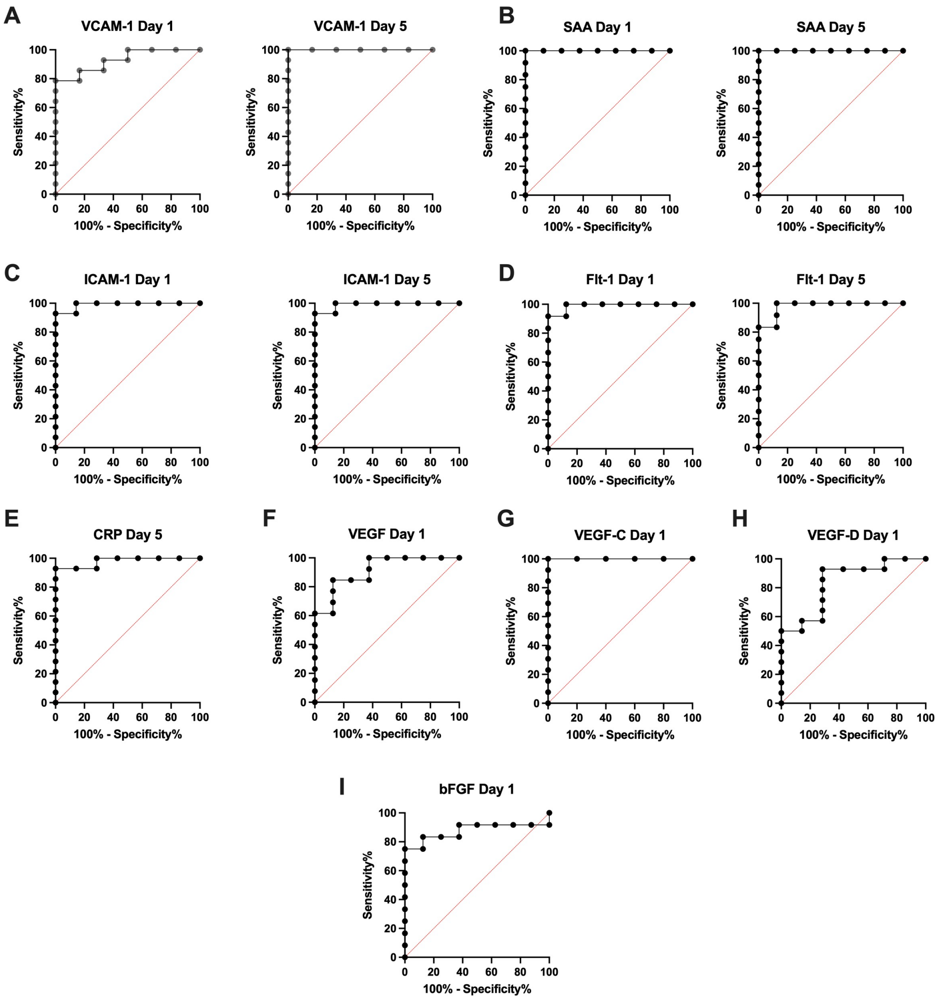

Supplementary Figure S1. ROC for Vascular Injury and Angiogenesis Biomarkers of aSAH in CSF. The ROC and AUC were calculated for **(A)** VCAM-1, **(B)** SAA, **(C)** ICAM-1, **(D)** Flt-1, **(E)** CRP, **(F)** VEGF, **(G)** VEGF-C, **(H)** VEGF-D, and **(I)** bFGF as they were previously identified to be significantly elevated in the CSF of patients with aSAH when compared to hydrocephalus controls. (**A**) VCAM-1: N: control: 6, aSAH: 14; **(B)** SAA: N: control: 8, aSAH: 12 **(C)** ICAM-1: N: control: 7, aSAH: 14; (**D)** Flt-1: N: control: 8, aSAH: 12; (**E**) CRP: N: control: 7, aSAH: 14; **(F)** VEGF: N: control: 8, aSAH: 13; **(G)** VEGF-C: N: control: 5 aSAH: 13; **(H)** VEGF-D: N: control:7 aSAH: 14; **(I)** bFGF: N: control: 8 aSAH: 12.

ROC CURVES: Serum

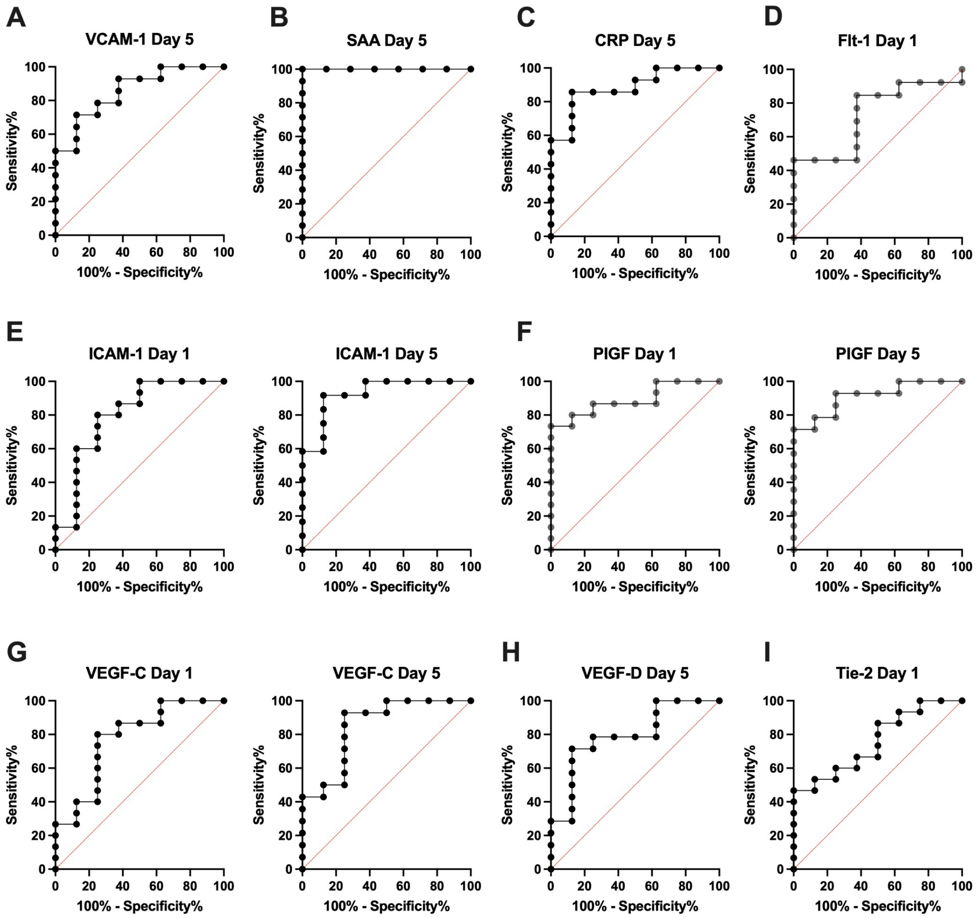

Figure S2. ROC for Vascular Injury and Angiogenesis Biomarkers of aSAH in Serum. The ROC and AUC were calculated for **(A)** VCAM-1, **(B)** SAA, **(C)** CRP, **(D)** Flt-1, **(E)** ICAM-1, **(F)** PlGF, **(G)** VEGF-C, **(H)** VEGF-D, and **(I)** Tie-2 as they were previously identified to be significantly elevated in the serum of patients with aSAH when compared to healthy, age-matched controls. (**A**) VCAM-1: N: control: 8, aSAH: 15; **(B)** SAA: N: control: 7, aSAH: 11; (**C**) CRP: N: control: 8, aSAH: 15; **(D)** Flt-1: N: control: 8, aSAH: 12; **(E)** ICAM-1: N: control: 8, aSAH: 15; **(F)** PlGF: N: control: 8, aSAH:15; **(G)** VEGF-C: N: control: 5 aSAH: 13; **(H)** VEGF-D: N: control:7 aSAH: 14; **(I)** Tie-2: N: control: 8, aSAH: 15.

ROC CURVES: Pro-Inflammatory Cytokines in Serum

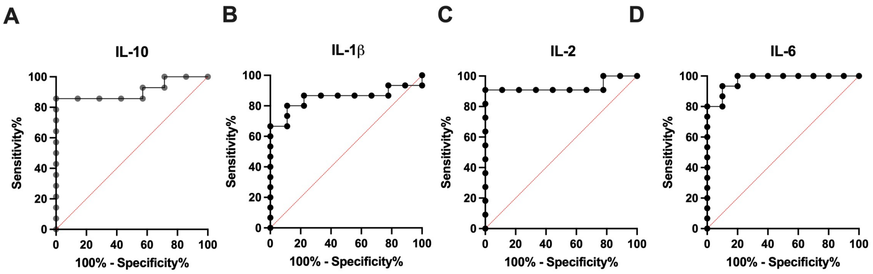

Figure S3. ROC for Pro-Inflammatory Cytokines of aSAH in Serum. The ROC and AUC were calculated for **(A)** IL-10, **(B)** IL-1β, **(C)** IL-2, and **(D)** IL-6. **(A)** IL-10: N: control: 7, aSAH: 14; **(B)** IL-1β: N: control: 9, aSAH:15; **(C)** IL-2: N: control: 9, aSAH: 11; **(D)** IL-6: N: control: 10, aSAH: 15.
